# Cortico-Striatal Oscillations Are Correlated to Motor Activity Levels in Both Physiological and Parkinsonian Conditions

**DOI:** 10.3389/fnsys.2020.00056

**Published:** 2020-08-13

**Authors:** Cristóbal Moënne-Loccoz, Carolina Astudillo-Valenzuela, Katrine Skovgård, Carolina A. Salazar-Reyes, Sebastian A. Barrientos, Ximena P. García-Núñez, M. Angela Cenci, Per Petersson, Rómulo A. Fuentes-Flores

**Affiliations:** ^1^Biomedical Neuroscience Institute, University of Chile, Santiago, Chile; ^2^Laboratorio de Control Motor y Neuromodulación, Facultad de Medicina, Universidad de Chile, Santiago, Chile; ^3^Department of Health Sciences, Faculty of Medicine, Pontificia Universidad Católica de Chile, Santiago, Chile; ^4^Programa de Doctorado en Ciencias Biomédicas, Facultad de Medicina, Universidad de Chile, Santiago, Chile; ^5^Department of Experimental Medical Science, The Group for Integrative Neurophysiology and Neurotechnology, Lund University, Lund, Sweden; ^6^Basal Ganglia Pathophysiology Unit, Department of Experimental Medical Science, Lund University, Lund, Sweden; ^7^Programa de Magíster en Neurociencias, Facultad de Medicina, Universidad de Chile, Santiago, Chile; ^8^Department of Integrative Medical Biology, Umeå University, Umeå, Sweden; ^9^Departamento de Neurociencia, Facultad de Medicina, Universidad de Chile, Santiago, Chile

**Keywords:** cortex, striatum, oscillations, local field potential, parkinsonian, motor activity, acceleration, beta

## Abstract

Oscillatory neural activity in the cortico-basal ganglia-thalamocortical (CBGTC) loop is associated with the motor state of a subject, but also with the availability of modulatory neurotransmitters. For example, increased low-frequency oscillations in Parkinson’s disease (PD) are related to decreased levels of dopamine and have been proposed as biomarkers to adapt and optimize therapeutic interventions, such as deep brain stimulation. Using neural oscillations as biomarkers require differentiating between changes in oscillatory patterns associated with parkinsonism vs. those related to a subject’s motor state. To address this point, we studied the correlation between neural oscillatory activity in the motor cortex and striatum and varying degrees of motor activity under normal and parkinsonian conditions. Using rats with bilateral or unilateral 6-hydroxydopamine lesions as PD models, we correlated the motion index (MI)—a measure based on the physical acceleration of the head of rats—to the local field potential (LFP) oscillatory power in the 1–80 Hz range. In motor cortices and striata, we observed a robust correlation between the motion index and the oscillatory power in two main broad frequency ranges: a low-frequency range [5.0–26.5 Hz] was negatively correlated to motor activity, whereas a high-frequency range [35.0–79.9 Hz] was positively correlated. We observed these correlations in both normal and parkinsonian conditions. In addition to these general changes in broad-band power, we observed a more restricted narrow-band oscillation [25–40 Hz] in dopamine-denervated hemispheres. This oscillation, which seems to be selective to the parkinsonian state, showed a linear frequency dependence on the concurrent motor activity level. We conclude that, independently of the parkinsonian condition, changes in broad-band oscillatory activities of cortico-basal ganglia networks (including changes in the relative power of low- and high-frequency bands) are closely correlated to ongoing motions, most likely reflecting he operations of these neural circuits to control motor activity. Hence, biomarkers based on neural oscillations should focus on specific features, such as narrow frequency bands, to allow differentiation between parkinsonian states and physiological movement-dependent circuit modulation.

## Introduction

Oscillatory neuronal activity and brain rhythms are thought to participate in key aspects of sensorimotor integration (MacKay, 1997). A way to measure neural oscillatory activity is to capture the low-frequency component of extracellular recording in brain structures, known as local field potential (LFP), which mainly reflects the synaptic activity in the recorded area (reviewed by Buzsáki et al., 2012). LFP from motor cortices and basal ganglia oscillates in different frequencies concerning motor control, including sensorimotor integration, preparation for voluntary movements, and oculomotor control (Murthy and Fetz, 1992; Sanes and Donoghue, 1993; Courtemanche et al., 2003).

Early findings showing increased basal ganglia oscillations in the low-frequency range (<30 Hz) in primate models of Parkinson’s disease (PD) led to the notion that these reflected pathological increases in neural synchronization (Bergman et al., 1994, 2001; Nini et al., 1995; Raz et al., 1996). This assumption was supported by the correlations found between parkinsonian motor signs and increased neural synchrony at low frequencies (Levy et al., 2000, 2002b; Costa et al., 2006), and by the observed decrease in low-frequency neuronal synchrony upon pharmacological treatments or electrical stimulation approaches that produced a symptomatic benefit (Brown et al., 2001; Levy et al., 2002a; Williams et al., 2002; Priori et al., 2004; Sharott et al., 2005; Marceglia et al., 2006; Eusebio et al., 2011).

Overall, these findings have led to the hypothesis that enhanced oscillations in the range of ~11–30 Hz, a range referred to as “beta band,” could be the causal link between dopamine loss and the emergence of motor signs in PD (Weinberger et al., 2009). A current view of this phenomenon is that beta oscillations are present physiologically in the cortico-basal ganglia-thalamocortical (CBGTC) loop as short-duration events named beta bursts and that these bursts are of greater duration and power in parkinsonian conditions, as seen in both dopamine-depleted animals and PD patients (Deffains and Bergman, 2018; Deffains et al., 2018).

Beta oscillations have been suggested as an electrophysiological biomarker for severity of parkinsonism that could be used as an input variable for adaptive or closed-loop controlled delivery of subthalamic deep brain stimulation for the treatment of PD (reviewed by Bouthour et al., 2019). On the other hand, in both healthy and parkinsonian individuals, changes in beta oscillations are observed during different motor states (Sanes and Donoghue, 1993; Brown et al., 2001; Courtemanche et al., 2003; Costa et al., 2006; Fuentes et al., 2009; Leventhal et al., 2012; Delaville et al., 2014). Thus, using beta oscillations as a biomarker for PD requires differentiating the changes in beta oscillations related to parkinsonism from those occurring physiologically upon motor state transitions. On a general level, it is of paramount importance to identify the impact of motor activity when studying oscillatory phenomena in the CBGTC loop.

The current study aims to examine how broad-frequency neural oscillations change according to the levels of motor activity in both intact and parkinsonian rats. To this end, we simultaneously recorded LFPs from motor cortices and striata and levels of motor activity while the animals were freely moving. A “motion index (MI)” was computed using signals collected from head-mounted accelerometers and correlated with the spectral power of the LFPs in the frequency band from 1–80 Hz.

## Materials and Methods

### Animals and Experimental Groups

Experiments were performed in adult Sprague–Dawley rats obtained from the central vivarium of the University of Chile Medical Faculty or approved suppliers (Janvier Labs, France). The study includes the following experimental groups and numbers of animals: (i) bilateral intrastriatal 6-hydroxydopamine (6-OHDA) injections (*n* = 5); (ii) bilateral intrastriatal injections of saline (*n* = 5); (iii) unilateral injections of 6-OHDA into the medial forebrain bundle (*n* = 9). Animals were housed in standard cages with *ad libitum* access to food and water and were kept on an inverted 12 h light/dark cycle. All experiments had received ethical approval by the appointed authorities (Bioethics Committee on Animal Research at the Faculty of Medicine, University of Chile, and Ethical Committee on Animal Research at Malmö-Lund Court District, Sweden).

### Bilateral 6-Hydroxydopamine Lesions and Electrode Implants

Surgeries for bilateral 6-OHDA lesions and chronic implant of recording electrodes were performed under deep isoflurane anesthesia (~1–1.5%). Non-steroidal anti-inflammatory drugs were given as post-surgical analgesia and prophylactic antibiotics were administered for 3 days after each surgery. After 6-OHDA lesions, rats were fed with peanut butter and sweetened milk until they were feeding normally again (within 4–7 days). After this period, rats were fed *ad libitum* with softened pellets.

6-Hydroxydopamine Hydrochloride (Sigma–Aldrich) was dissolved in ascorbate-saline at the concentration of 3.3 μg/μl (free base) according to published protocols (Cenci and Lundblad, 2007). Bilateral lesions (*n* = 5) consisted of three injections of 6-OHDA into each side of the striatum (2 μl/per injection, 20 μg 6-OHDA per side). These injections coordinates were used (in mm relative to Bregma and the dural surface): (1) AP 1.0, ML ± 3.0, DV −5.0; (2) AP −0.1, ML ± 3.7, DV −5.0; (3) AP −1.2, ML ± 4.5, DV −5.0. The toxin solution was infused at the rate of 1 μl/min and the needle was then left in place for 5 min to minimize backward diffusion along the injection tract. Control animals (*n* = 5) received the same volume of an ascorbate-saline solution without 6-OHDA. All animals receive a dose of desipramine (25 mg/kg, i.p, Sigma–Aldrich) 30 min before surgery to protect noradrenergic neurons.

At least 2 weeks following lesion surgery, an array of custom-made tungsten recording electrodes was implanted. Nine electrodes were directed to each primary motor cortex—caudal forelimb area (CFA/M1; Neafsey et al., 1986) and seven to each dorsolateral striatum (DLS). Center coordinates were: CFA/M1, AP: + 1.2, ML: ± 2.5, DV: −1.4; DLS, AP: + 0.35, ML: ± 3.35, DV: −2.6. Silver wire on the electrode array was attached to screws in the frontal and occipital skull bone for ground connection, and the implant was anchored to the screws in the skull with dental acrylic covering the ground wires for electrical insulation.

### Unilateral 6-OHDA Lesions and Electrode Implants

Surgeries for unilateral 6-OHDA lesions and implantation of chronic recording electrodes were performed under fentanyl/medetomidine anesthesia (0.21/0.21 mg/kg, i.p.; Apoteket AB, Sweden). After surgery, the anesthesia was reversed by atipamezole hydrochloride (0.5 mg/kg, i.p.; Apoteket AB, Sweden) and buprenorphine (0.05 mg/kg, s.c.; Apoteket AB, Sweden) was administered as a postoperative analgesic. After the surgeries, animals were fed with softened pellets and peanut butter for approx. 1 week before resuming the standard feeding regimen.

6-Hydroxydopamine Hydrochloride (Sigma–Aldrich) was dissolved, handled, and infused as described above. To obtain unilateral lesions, 6-OHDA was injected into the right medial forebrain bundle (MFB) using the following coordinates:1st injection (2.5 μl), AP −4.0, ML −1.2, DV −7.8, tooth bar in flat skull position (approximately TB −4.5); 2nd injection (2.0 μl), AP −4.0, ML −0.8, DV −8.0, tooth bar +3.4.

Three weeks after lesion, animals with severe (>85%) unilateral dopamine denervation were selected using the cylinder test of forelimb use asymmetry (Lundblad et al., 2002; Francardo et al., 2011; Sebastianutto et al., 2016). Briefly, rats were placed individually in a glass cylinder (25 cm in diameter and 40 cm in height) and video filmed for 3 min. Supporting wall contacts performed independently with the left and right forepaw were counted offline. Counts from the forelimb contralateral to the lesion (left) were expressed as a percentage of the total number of wall contacts, and rats with more than 25% contralateral paw usage were excluded from the study.

Electrode implantation surgeries were performed at least 6 weeks after 6-OHDA lesions. An array of tungsten microwire electrodes (33 μm, California Wires) was prepared as previously described (Ivica et al., 2014). The electrodes were connected to a custom-designed Printed Circuit Board (PCB) on each hemisphere and secured with epoxy resin. In each hemisphere, the following cortical and striatal coordinates were targeted: rostral forelimb area (RFA; Neafsey and Sievert, 1982; AP 3.75, ML ± 2.0, DV −1.0), the forelimb area of the primary motor cortex (M1; Gioanni and Lamarche, 1985; AP 1.76, ML ± 2.71, DV −1.0), and the DLS (AP 0.11, ML ± 4.07, DV −4.0; center coordinates are given in mm relative to bregma and cortical surface; tooth bar in flat skull position). Silver wire on the electrode array was attached to screws in the frontal and occipital skull bone for ground connection, and the implant was anchored to the skull screws with dental acrylic cement covering the ground wires for electrical insulation. After surgery, the animals were allowed to recover for a minimum of 1 week before the recordings commenced.

### Recordings

Bilaterally lesioned animals were placed in a transparent box of 32 × 25 × 30 cm (length × width × height) to acquire both electrophysiology/acceleration data and video recordings for 20 min on three different days. Although the rats could move freely throughout the recording time, during the first 10 min motor activity was encouraged using objects and toys that the rats could explore.

Unilaterally lesioned animals were placed in a transparent cylinder (diameter 55 cm, height 40 cm) and recorded for both electrophysiology/acceleration and video data for 20 min in two or three different days.

### Signal Acquisition

Electrophysiological recordings were performed with an RHD2000-Series Amplifier Evaluation System (Intan Technologies, LLC, California, CA, USA) and the software Open Ephys GUI. To obtain the LFPs, signals from unfiltered channels (referenced to three electrically connected skull screws located in the occipital and frontal bone) were digitized at 30 kHz and saved.

### Video Recordings

Video recordings were obtained using a Flea2 FireWire 1394b camera (Point Gray) at a rate of 15 frames per second (fps) or a Genie HM640 camera at 25 fps, resolution of 640 × 480 pixels, and positioned at 85 cm or 150 cm respectively from the floor of the box (top view of the box). Synchronization between accelerometer signal and video frames was performed using an Arduino system, which simultaneously lit a LED while sending an analog TTL to the Intan recording system or, in the case of the Genie camera, a TTL pulse triggered each frame capture, as well as a copy of it, was sent as a digital input through a splitter to the Intan recording system. The frame-by-frame position of the rats in the open field plane was obtained by off-line processing the videos with the open-source software Bonsai (Lopes et al., 2015). Then these positions were converted to instant speed by taking the difference of the rat position between two contiguous frames and dividing them by the time interval between the frames (15 fps = 0.067 s, 25 fps = 0.04 s).

### Spectral Power Analysis

Raw LFP recordings were down-sampled offline from 30 kHz to 1,000 Hz. Each channel was then re-referenced by subtracting the average signal of all the channels belonging to the same structure. Channels with poor recording quality were excluded before re-referencing. Processed LFP was divided into epochs of 1-s duration in steps of 0.5 s. Epochs were submitted to an automatic artifact rejection of peak-to-peak of 1 mV and flat signal detection. Epochs with artifacts or flat signals were discarded from further analysis. Then, for each epoch, we calculated the power spectral density (PSD) using the multitaper method in the frequency range 0.5–80 Hz in intervals of 1 Hz. Specifically, we used multiple discrete prolate spheroidal sequences (DPSS) tapers with a half-bandwidth window of 2 Hz (three tapers). The PSD values were then transformed to decibels (dB) by applying natural logarithm multiplied by 10. For each brain area per subject/hemisphere, a representative PSD was obtained by averaging all individual channels PSD from the same area (using 2–9 channels per area).

### Acceleration Recordings

Acceleration signals were recorded with a sampling rate of 30 kHz using two analog 3-axis ADXL335 accelerometers, each located in the Intan recording headstages (Intan RHD2132) connected to the chronic electrode implant of the rats. Accelerometers were calibrated before the recording sessions and sensed both movement and orientation concerning gravity. Offline, the data of both sensors were downsampled to 1,000 Hz and averaged in each x/y/z axes to obtain a single acceleration measure from each animal.

### Motion Index

Following a similar approach as reported by Oza et al. (2018), we quantified the motor activity of the animals by computing a motion index based on the dynamic acceleration. First, the raw accelerometer signals were divided into epochs into the corresponding LFP intervals of 1 s in steps of 0.5 s. Next, we calculated the average PSD of each axis in the frequency range 1–45 Hz using the same approach mentioned in spectral power analysis. Note that this frequency interval includes the global characteristics of the movement of the animal’s head (where the accelerometers were located), without the gravitational component also measured with the accelerometers (da Silva et al., 2018). Then, we averaged the total 1–45 Hz spectral power density over the three axes to obtain a single value per epoch, the motion index. To compensate for the subtle differences in the positions of the accelerometers in each animal, we normalized the individual motion index values to a common space. First, we performed a kernel density estimation (KDE) of the probability density function of the motion index values with a Gaussian kernel of bandwidth selected using Scott’s rule (Scott, 1992). We used this peak as a normalization value, which was subtracted from the individual motion index values. Thus, the first peak of the density was always positioned at the motion index zero for each animal. The unit of the motion index is expressed in dB, however, since it was referenced to a common zero, the unit is omitted in the following text, and referred to as arbitrary units (AU) in the figures.

### Motion Index Bin Analysis

To equalize bin count without losing finer granularity of the movement states and to maintain the same variance between bins, we defined a non-uniform binning procedure. To define the bounds of each bin, starting from the minimum value of the motion index as the lower bound, we iteratively adjusted the bin size until it had 100 samples. The representative value for each motion index bin was defined as the middle point between the bins bounds. The average bin size obtained through this procedure was 0.1 ± 0.1 (mean ± standard deviation) motion index units, while the average number of bins was 586 ± 334 (mean ± standard deviation). Finally, to avoid large bin sizes at the edges of the distributions, we limited the minimum/maximum value such that the first/last bin size was not greater than 0.5 motion index units. To grand average the data of the different animals, the bin data was interpolated in a uniform grid of 150 points between normalized motion indexes from −5 to 25.

### Unimodality Test

To analyze the overall shape of the normalized motion index distribution and the speed distribution, we used the Hartigan Dip-test, which “*measures multimodality in a sample by the maximum difference, over all sample points, between the empirical distribution function, and the unimodal distribution function that minimizes that maximum difference*” (Hartigan and Hartigan, 1985).

### Correlation Analysis

To correlate the motion index with the binned power of each frequency, we used Spearman’s rank correlation coefficient, denoted by the Greek letter ρ (rho). This coefficient compares monotonically relationships between two variables whose relationship is not necessarily linear. The correlation values vary between −1 and 1, being −1 anti-correlation and 1 perfect correlation.

### Tyrosine Hydroxylase Immunostaining

Animals were deeply anesthetized with ketamine and xylazine (100 mg/kg and 10 mg/kg, respectively) and transcardially perfused with 0.9% saline followed by 4% buffered paraformaldehyde (PFA) solution. The brains were removed and post-fixed in 4% PFA for 24 h at 4°C, then kept in phosphate-buffered saline (PBS) containing 30% sucrose at 4°C. Brains were cut coronally into 30 μm thick sections using a cryostat.

Free-floating brain sections were processed for tyrosine hydroxylase (TH) histochemistry using an established protocol (Francardo et al., 2011) with minor modifications. Briefly, following a peroxidase-quenching step (0.3% H_2_O_2_ for 30 min), sections were incubated for 1 h at room temperature (RT) in phosphate-buffered saline (PBS) containing 0.5% bovine serum albumin (BSA) and 0.2% Triton X-100. Then, sections were incubated with an anti-TH antibody (Merck, Cat # Ab152; 1:1,000) overnight at 4°C. On the following day, after rinses in PBS, sections were incubated with the secondary biotinylated antibody for 2 h at RT, followed by Vectastain ABC-peroxidase complex (Vector Labs, cat PK-4001) according to the manufacturer’s instructions.

Finally, a color reaction was developed using 3,3′-diaminobenzidine (Sigma Cat# D5905) in Tris buffer (Sigma Cat# T5030) for 4–5 min. Slide-mounted sections were digitized using an Epson L355 scanner (eight bits, 4,800 dpi resolution). Optical density measurements were taken on 6–12 striatal sections per animal using the ImageJ software^1^. The percentage loss in 6-OHDA lesioned striata was determined concerning sham-lesioned rats. In bilaterally lesioned rats, the average loss of striatal TH immunoreactivity was 79.8 ± 11.5% (mean ± SD). Unilaterally lesioned animals showed >85% loss of TH immunostaining on the affected side.

## Results

To compare the relationship between oscillations and motor activity in normal vs. parkinsonian conditions, we used both bilaterally and unilaterally 6-OHDA-lesioned rats as the two variations of the rodent PD model that is most widely used for pathophysiological research (Cenci and Crossman, 2018). While rats were freely moving in an open field, we recorded the LFPs from motor cortices and striatum, the acceleration of head translocations using inertial sensors, and rat body position using video cameras.

### Accelerometers Provide Sensitive Measures of Motor Activity Level

**Figure 1:**
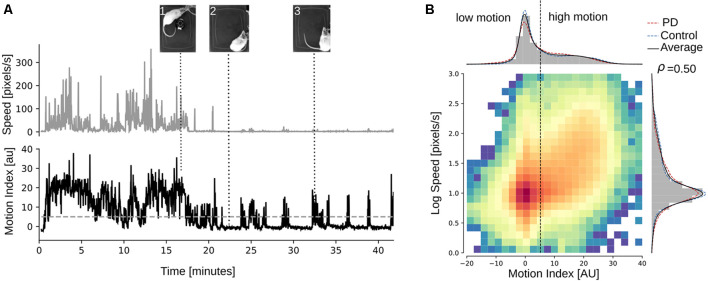
Accelerometry-derived motion-index (MI) detects motor activity accurately and is positively correlated to the speed of the rat. **(A)** Example of the speed (top panel) and the motion-index (bottom panel) of a bilateral 6-hydroxydopamine (6-OHDA) lesioned rat for 40 min. The pictures on top correspond to the rat in the open field at the moment indicated by the dotted lines. In the first half of the session (0–20 min approx.), the rat is navigating the open field, which is apparent in both the speed and the motion-index traces. But in the second half of the recording, according to the speed trace, the rat is not moving in the open field, but it is not entirely still, as can be observed in the pictures 2 and 3, and importantly, in the motion-index trace, which detects the exact moments when the rat perform head or body movements in place which do not result in navigating in the field. The gray dotted line represents the threshold for low/high motion-index as established next. **(B)** At the center, we show the 2D histogram of the speed in logarithmic scale (y-axis) and the motion-index (x-axis) generated by normalizing the distributions of each animal by aligning the first peak of the motion index (peak = −162.02 ± 6.02, mean ± standard deviation) to zero and the peak of log speed (peak = 0.7 ± 0.34) to one, for all the animals in the sample (*n* = 10 bilateral rats). The color scale of the histogram is normalized to a logarithmic scale to better visualize the positive correlation (Spearman’s ρ= + 0.50). At top and right, normalized histograms and Gaussian kernel density estimation (KDE; Scott’s rule bandwidth = 0.1) for both speed (right) and motion-index (top). Notice the bimodal distribution of the motion index, which can be separated in low/high states approximately at normalized index value 4.2. In contrast, the distribution of speed is unimodal, concentrating its mass mainly in the center of the log speed distribution. The distributions separated by 6-OHDA and sham are also included (dotted red and blue traces).

In this study, the term motion index refers to the average spectral power density in the range 1–45 Hz of the signals obtained from three orthogonal sensors detecting translational acceleration, located on the rat’s head. The instant speed of rat motions was obtained by deriving the body position in time from sequential video frames. A representative example of the motion index and speed recorded during a single session is shown in Figure 1A. While motion index and speed are correlated, the motion index can report motor activity when it is performed with low or no actual navigation in the open field (Figure 1A, see inset 3 and the corresponding speed and motion index). We found that the motion index was positively correlated to the speed of the rat (Spearman’s *ρ* = 0.50, *p* < 0.001, Figure 1B center), indicating that motion index values were greater, as expected, for higher locomotion speed in the field. The distribution of the motion index was not unimodal (Dip-test of unimodality, dip statistic = 0.039, *p* = 0.005, Figure 1B top), hence we used the two modes to divide the range of motion index values and define two distinct motor states: low- and high motion, with low motion associated to a state of minimum or no motor activity, while high motion corresponds to movement, but not necessarily actual navigation in the field. To determine the limits of the low and high motion states, a bimodal Gaussian adjustment to the histogram was used, leaving as a limit three standard deviations of the first peak (*MI* = 0) as the cutoff value between the states (three sigmas = 4.2 MI). Such classification could not be done with speed, as its distribution was found to be unimodal (dip statistic = 0.027, *p* = 0.24, Figure 1B right).

#### Sorting LFP Power Spectral Density According to Motion Index Results in Distinctive Oscillatory Patterns

**Figure 2:**
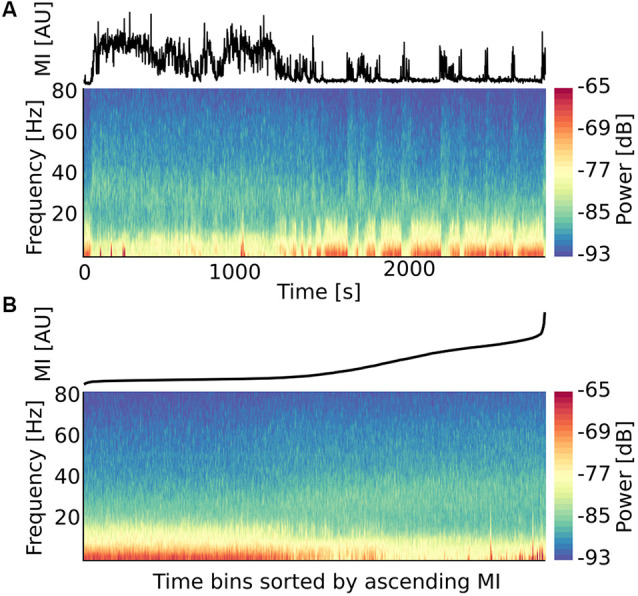
Representation of a spectral power chart as a function of *motion index* reveals a tight correlation between motor state and oscillations at specific frequency bands. **(A)** Example of a time-frequency-power chart computed from local field potential (LFP) signals from the right motor cortex of a bilateral 6-OHDA rat extended for 40 min with the corresponding motion index. **(B)** The same chart but sorted according to the ascending motion index.

To study the relationship between the motor state and neural oscillatory activity, we correlated the motion index and the LFP power at different frequencies. To this end, the representation of LFP power spectral density as a function of time (Figure 2A) was complemented with representations of the corresponding power bins sorted in ascending order (left to right) concerning the concomitantly measured motion index (Figure 2B). Such an arrangement revealed a neat relationship between spectral broad-band power changes and motor activity levels.

**Figure 3:**
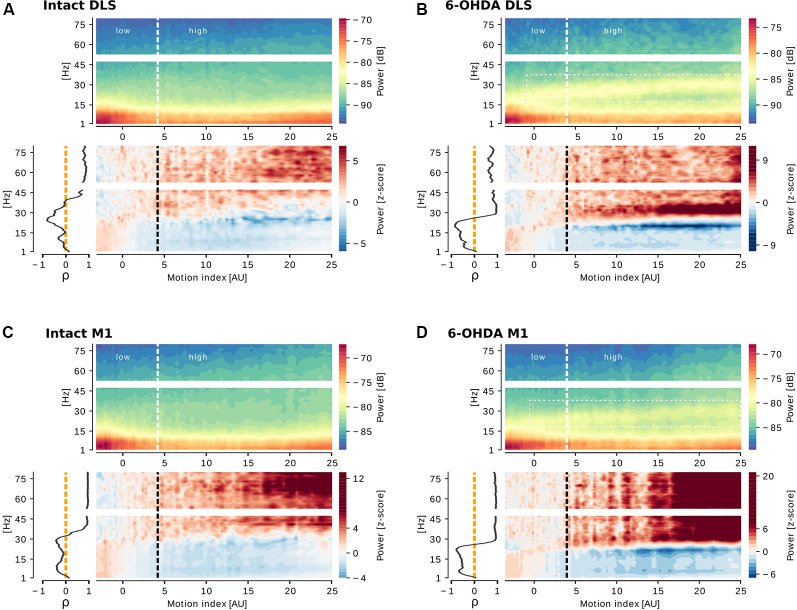
Cortico-striatal oscillations are correlated to motor activity levels in the intact and lesioned hemisphere of unilateral 6-OHDA rats. For each panel, motion index (MI)-frequency charts of power spectral density (PSD) in decibels (dB; top) or z-score (bottom right), and Spearman’s correlation (ρ) between power and frequency (bottom left) are shown. At low MI, an increase was seen in power below ~30 Hz, while the power above that frequency increases with increasing MI in the dorsolateral striatum (DLS) and primary motor cortex (M1) of the intact (**A** and **C**, respectively) or lesioned (**B** and **D**, respectively) hemisphere of a unilateral 6-OHDA rat. Spearman’s correlation confirms a negative correlation between MI and LFP oscillations below ~40 Hz and a positive correlation above that frequency. The 6-OHDA injected areas present a distinctive narrow band in the range ~25–40 Hz (**B** and **D**, white rectangle) which frequency peak shifts as MI increases.

For a detailed examination of the motor activity-dependent changes in LFP power according to the 6-OHDA lesion model (unilateral or bilateral injection), motion index-frequency charts were computed for all groups and brain areas. Figure 3 shows representative examples of motion index-frequency charts, with spectral power represented either as decibels (Figure 3, top of each panel) or z-score (Figure 3, the bottom of each panel) relative to low-motion of each brain area in the intact and 6-OHDA lesioned hemispheres of 6-OHDA unilateral rats. The z-scored charts reveal that low-frequency power is increased at low motion index values, and that high-frequency power is increased at high motion index values in the striata and the motor cortices of both intact (Figures 3A,C, bottom charts) and dopamine-denervated hemispheres (Figures 3B,D, bottom charts). Also, to quantify the motion index—power correlation, Spearman’s rank-order correlation was computed for every frequency-power series against the motion index and represented as Spearman’s ρ vs. frequency (left chart of every panel of Figure 3). This test revealed a negative correlation of power with motion index at low frequencies (< ~30 Hz) and a positive correlation with power at high frequencies (> ~40 Hz) in striata and motor cortices of intact (Figures 3A,C, left panel) and 6-OHDA-lesioned hemispheres (Figures 3B,D, left panel).

While this analysis revealed a common feature between physiological and parkinsonian conditions relative to the motor activity levels and oscillations in broad frequency bands, a difference in a characteristic narrow band (25–40 Hz) was apparent in the lesioned striata and cortex of the unilateral rats (Figures 3B,C, top panels). This narrow frequency band appears only during walking and has previously been described in the substantia nigra, subthalamic nucleus, and cortex of the lesioned hemisphere of unilaterally injected 6-OHDA rats (Avila et al., 2010; Delaville et al., 2015). Remarkably, the sorting of the oscillations according to increased motion index revealed a novel characteristic of these oscillations, which is the shift from lower (~20 Hz) to higher (~30 Hz) frequencies as the motion index increases.

**Figure 4:**
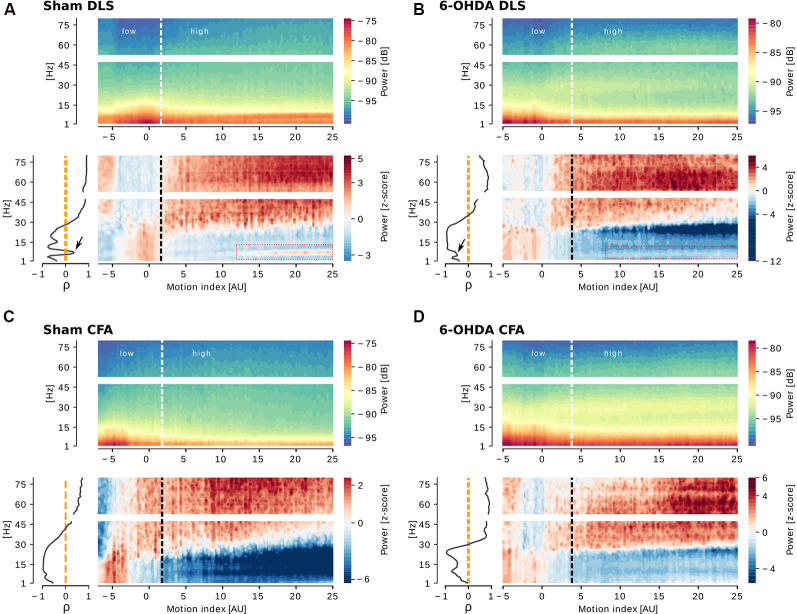
Cortico-striatal oscillations are correlated to motor activity levels in sham and 6-OHDA bilateral rats. For each panel, motion index (MI)-frequency charts of PSD in dB (top) or z-score (bottom right), and Spearman’s correlation between power and frequency (bottom left) are shown. The common feature of increased low-frequency power at low MI and increased high-frequency power at high MI is observed in the dorsolateral striatum (DLS) and caudal forelimb area (CFA) of a sham (**A** and **C**, respectively) and an injected (**B** and **D**, respectively) bilateral 6-OHDA rat. The DLS of the sham and the 6-OHDA bilateral rats present a narrow band with a peak at ~7.5 Hz (red rectangles in **A** and **B**) which power is positively correlated to MI.

Figure 4 is analogous to Figure 3 but shows representative examples of the correlation between LFP oscillations and motion index in brain areas from sham and 6-OHDA lesioned bilateral rats. The same features regarding broad frequency oscillations are present in this case in both physiological and parkinsonian brains: increased low-frequency oscillations at low motion index and increased high-frequency oscillations at high motion index (Figures 4A–D, bottom charts). Yet, a narrow frequency band exhibits distinctive features. This band, with a peak at 7.5 Hz, showed less negative, or even positive, correlation to motion index than the surrounding broad-band (representative examples in the ρ-frequency plots in Figures 4A,B, left panel). This 7.5 Hz narrow band is also apparent in the z-scored motion index-frequency chart (Figures 4A,C, bottom charts), where it shows increasing power in high motion. The 7.5 Hz band was observed in 5/5 bilateral 6-OHDA rats, 4/5 sham bilateral rats, and in 5/9 unilateral rats (1/9 only in the intact hemisphere, 2/9 only in the 6-OHDA injected hemisphere, and 2/9 in both intact and injected hemispheres).

#### Striatal and Cortical Broad-Band LFP Oscillations Are Correlated to Motor Activity in Physiological and Parkinsonian Conditions

To precisely identify the frequency bands of the oscillations correlated to motion, we computed the grand mean for every brain area/condition group, confirming the findings of a stereotyped correlation between corticostriatal broad-band oscillations and levels of motion, regardless whether the brain is parkinsonian or not (Supplementary Figure S1). Thus, considering all areas and conditions together (*n* = 19 rats) in the frequency range analyzed (1–80 Hz), we identified two broad-bands correlated to movement: a low-frequency band negatively correlated to motion index (Spearman’s *ρ* < −0.5) between 5.0 ± 5.2 and 26.5 ± 7.4 Hz (median ± median absolute deviation), and a high-frequency band positively correlated to motion index (Spearman’s *ρ* > 0.5) between 35.0 ± 10.4 and 79.9 ± 0 Hz.

**Figure 5:**
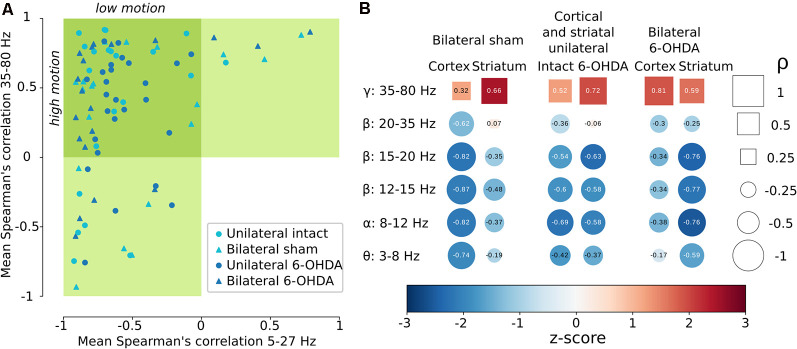
The correlation between LFP oscillatory activity and movement is preserved in physiological and parkinsonian conditions. **(A)** Scatter plot of the average correlation in the range 5–27 Hz (x-axis) and 35–80 Hz (y-axis). Each marker represents a brain area (cortex or striatum) of a single rat. 60 out of 86 (70%) data points are in the quadrant corresponding to negative correlation with low-frequencies and positive correlation with high-frequencies. **(B)** Summary of the correlations found for classic frequency bands and brain areas. Circles and squares are for negative and positive correlation, respectively. The relative size of the circles and squares and the values within represent the magnitude of the correlations. The colors represent the average PSD during high-motion expressed as the z-score relative to the low-motion segment.

To get an overall view of the outcome in the different experimental groups concerning the oscillation/motion correlation, the different areas of each rat were represented as coordinates in 2-dimensional space using the average correlation in each of the two bands previously identified. The outer bounds of the bands were established by the rounded median value, thus resulting in 5–27 Hz for low-frequency and 35–80 Hz for high-frequency. This analysis revealed that most of the data points, 59 out of 86 (69%), were located in the quadrant that corresponds to a negative correlation to low-frequency and positive correlation to high-frequency (Figure 5A). Of these, 34 (58%) are 6-OHDA-lesioned areas and 25 (42%) are non-dopamine-denervated areas. These results again support that the correlation between the oscillatory power of both cortical and striatal LFPs and motion is preserved in both physiological and parkinsonian conditions.

Finally, to summarize the magnitude of the correlation between spectral power and activity state in terms of the frequency bands described in the literature, we represented the correlation and the power during high-motion (Figure 5B). This display confirms the general feature of anti-correlation of the cortical and striatal oscillations at frequencies <35 Hz (theta, alpha, and beta), as they dominate during low motion and are diminished during high-motion (light and dark blue circles in Figure 5B). In contrast, oscillations >35 Hz (gamma), which are positively correlated to movement, are more powerful during high motion (light and dark red squares in Figure 5B) and weakened during low motion.

## Discussion

Current electrophysiological research on neural motor control has incorporated the use of inertial sensors, including accelerometers, for precise assessment of movement kinematics (Venkatraman et al., 2010; Klaus et al., 2017; da Silva et al., 2018). In this study, we used head-mounted accelerometers to quantify motor activity levels at high temporal resolution and then sorted the simultaneously obtained LFP oscillations from the motor cortex and striatum based on this data.

Sorting cortical and striatal oscillations according to increasing motor acceleration revealed that broad-band oscillatory neural activity is robustly correlated to motor activity levels. In particular, the power of low-frequency oscillations in the bandwidth 5–27 Hz is higher during states of immobility or minimal motor activity, whereas the high-frequency band 35–80 Hz is more prominent during active motor states. This dual correlation was observed in both controls and dopamine-denervated rats sustaining unilateral or bilateral dopaminergic lesions.

The 5–27 Hz band covers several physiological relevant frequency bands such as theta (6–9 Hz). We observed a narrow band with a peak at 7.5 Hz that was correlated to increased motor activity, in opposition to the correlation to immobility of the 5–27 Hz broad-band. The frequency of this narrow band falls within the theta range. Oscillations in such range are found in the hippocampus and are correlated to locomotion in CA1 (Fuhrmann et al., 2015; but also see Lalla et al., 2017). While there are no hippocampal projections to the dorsal striatum, functional connectivity and interactions in the theta band in the context of different behaviors have been described for this pair of brain structures in rats (DeCoteau et al., 2007; Tort et al., 2013) and humans (Ross et al., 2011; Herweg et al., 2016), suggesting that the theta oscillations observed in our study might be related to the hippocampal activity.

Another relevant physiological band contained within the motor activity anti-correlated 5–27 Hz broad-band is the beta band (12–35 Hz). Increased beta oscillations of LFPs in the cortex and basal ganglia nuclei have been consistently reported from PD patients (reviewed in Brown, 2003) and dopamine-denervated animals (reviewed by Halje et al., 2019), suggesting that such oscillations could be used as a neurophysiological biomarker for therapeutic circuit modulation (Petersson et al., 2020). On the other hand, in neural motor circuits, beta oscillations have been regarded as playing a physiological role in motor control. The *status quo* hypothesis by Engel and Fries (2010) proposes that beta oscillations are related to the maintenance of the ongoing motor state. In fact, in the intact cortico-basal ganglia circuits of a behaving rat, beta power is enhanced once a motor response has been selected but yet not executed, suggesting that beta reflects a stable state of the neural circuit preventing interference of potential alternative motor responses (Leventhal et al., 2012). From a general electrophysiological perspective, increased low-frequency oscillations (beta for example) are thought to arise from increased synchrony of synaptic activity (Buzsáki et al., 2012). According to the information theory, a highly synchronized neuronal population carries substantially less information compared to the desynchronized state (Hanslmayr et al., 2012). In this scenario, the increased amplitude or duration of beta oscillations in parkinsonian conditions (Santana et al., 2014; Deffains et al., 2018) may reflect an over-stabilization of the basal ganglia neural circuit into a state that prevents the proper processing of cortical information to initiate volitional movements.

We could also readily identify the 25–40 Hz high beta oscillations previously described as selectively increased in the dopamine-denervated substantia nigra pars reticulata, cortex, and subthalamic nucleus in hemiparkinsonian rats (Avila et al., 2010; Delaville et al., 2014, 2015). These oscillations have been described to occur in a fixed frequency range, yet, sorting of oscillations according to motion index, as in our study, suggest that the actual frequency is related to the level of motor activity, starting in the lower limit at lower motor activity and increasing as the activity increases. The fact that in the original studies (Avila et al., 2010; Delaville et al., 2014, 2015), recordings were made in rats walking on a treadmill at a fixed speed, whereas we here considered all types of activity (ranging from subtle head movements to actual locomotion) probably explains why we are the first to observe that high beta frequency is positively modulated when motor activity levels increase.

The high-frequency band in 35–80 Hz, known as gamma oscillations, was correlated with increased motor activity. This observation is consistent with evidence showing increased gamma in cortical and basal ganglia structures in rodents when they start locomotion (Costa et al., 2006; Fuentes et al., 2009).

Overall, our results are compatible with the notion that neural motor control involves modulation of defined oscillatory patterns at different frequencies that are preserved in the parkinsonian state. We report that the power of low-frequency vs. high-frequency broad-band oscillations changes in opposite directions when motor activity increases or decreases, possibly reflecting different operational states of neural motor control systems. Whether the enhanced low-frequency oscillations observed in PD are causally linked or simply correlated to diminished motor output is still a matter of study (Little and Brown, 2014). Although important advances (Deffains et al., 2018) have been made since the time beta was considered a “bad oscillation” (Brown, 2006), these results highlight the relevance of developing more sophisticated algorithms to differentiate physiological from pathological oscillations in different motor states.

## Data Availability Statement

The raw data supporting the conclusions of this article will be made available by the authors, without undue reservation.

## Ethics Statement

The animal study was reviewed and approved by Bioethics Committee on Animal Research at the Faculty of Medicine, University of Chile; and Ethical Committee on Animal Research at Malmö-Lund Court District, Sweden.

## Author Contributions

CM-L, CA-V, MC, PP, and RF-F designed the experiments. MC provided input to study design and methods related to the animal models. CA-V, KS, CS-R, SB, and XG-N performed the experiments. CM-L analyzed the data. RF-F and PP wrote the manuscript with critical input from MC.

## Conflict of Interest

The authors declare that the research was conducted in the absence of any commercial or financial relationships that could be construed as a potential conflict of interest.

## Footnotes

^1^https://imagej.nih.gov/ij/
