# Supplementary material for: Cortico-Striatal Oscillations Are Correlated to Motor Activity Levels in Both Physiological and Parkinsonian Conditions
Source: Front Syst Neurosci. 2020 Aug 13;14:56. doi: 10.3389/fnsys.2020.00056 (PMC7439091; doi:10.3389/fnsys.2020.00056)
Supplement: Supplementary file 1 [file Image_1.pdf]

Supplementary Material

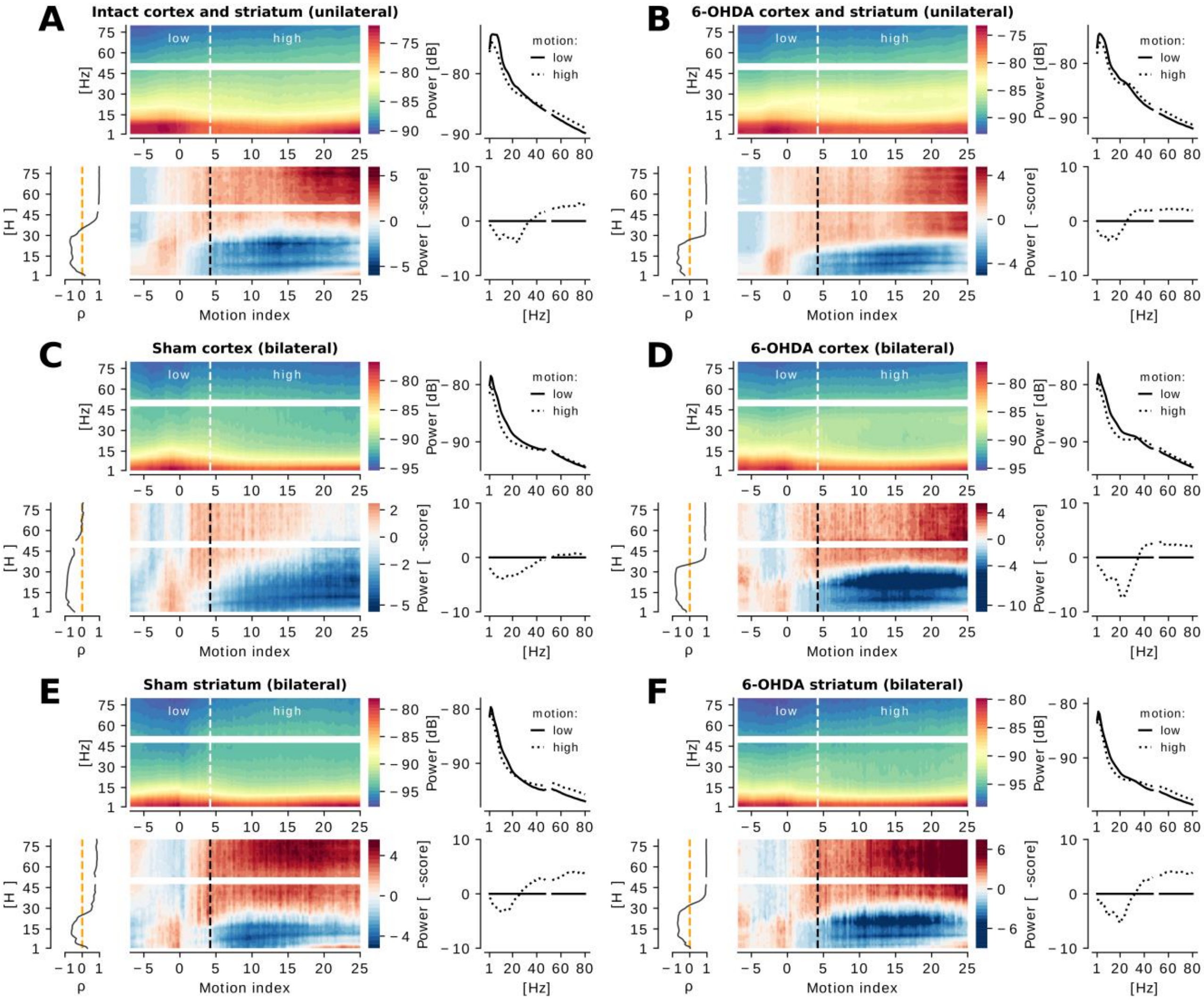

Supplementary Figure S1. Cortical and striatal LFP oscillations <30 Hz are anti-correlated to movement, while those in the range 30-80 Hz are correlated. Each panel presents the grand mean of the specified group. (A) Intact cortex and striatum of unilateral 6-OHDA lesioned rats (n=21 areas from 9 rats); (B) 6-OHDA injected cortex and striatum of unilateral lesion rats (n=26 areas from 9 rats); (C) motor cortex, bilateral 6-OHDA sham rats (n=19 areas from 5 rats); (D) motor cortex, bilateral 6-OHDA rats (n=26 areas from 5 rats); (E) DLS, bilateral sham rats (n=19 areas from 5 rats); (F) DLS, bilateral 6-OHDA rats (n=26 areas from 5 rats).
